# Non-motor asymmetry and dopamine degeneration in Parkinson’s disease

**DOI:** 10.1093/braincomms/fcaf002

**Published:** 2025-01-06

**Authors:** Frederik O Hansen, Karoline Knudsen, Malene F Damholdt, Toke Bek, Per Borghammer, Niels Okkels

**Affiliations:** Department of Nuclear Medicine and PET, Aarhus University Hospital, Aarhus N, 8200 Aarhus, Denmark; Department of Nuclear Medicine and PET, Aarhus University Hospital, Aarhus N, 8200 Aarhus, Denmark; Department of Clinical Medicine, Aarhus University Hospital, Aarhus N, 8200 Aarhus, Denmark; Department of Ophthalmology, Aarhus University Hospital, Aarhus N, 8200 Aarhus, Denmark; Department of Nuclear Medicine and PET, Aarhus University Hospital, Aarhus N, 8200 Aarhus, Denmark; Department of Clinical Medicine, Aarhus University Hospital, Aarhus N, 8200 Aarhus, Denmark; Department of Clinical Medicine, Aarhus University Hospital, Aarhus N, 8200 Aarhus, Denmark; Department of Neurology, Aarhus University Hospital, Aarhus N, 8200 Aarhus, Denmark

**Keywords:** Lewy body disease, synucleinopathy, laterality, lateralization, asymmetry

## Abstract

Asymmetric dopaminergic degeneration of the striatum is a characteristic feature of Parkinson’s disease, associated with right–left asymmetry in motor function. As such, studying asymmetry provides insights into progressive neurodegeneration between cerebral hemispheres. Given the impact of Lewy pathology on various neurotransmitter systems beyond the dopaminergic, it may be that other neuronal systems in the predominantly affected hemisphere are similarly affected. According to this hypothesis, asymmetry in dopaminergic degeneration would be expected to coincide with asymmetry in other neurotransmitter systems. Consequently, asymmetry in functions primarily dependent on dopaminergic integrity, such as motor function, should correlate with asymmetry in bilateral non-motor functions that rely on other cerebral systems, such as pupillary function. Therefore, this study tested whether right–left asymmetry in bilateral non-motor measures correlates with asymmetry in dopaminergic striatal integrity. We also tested whether asymmetric striatal degeneration is associated with greater asymmetry in non-motor measures overall. Using a comparative cross-sectional design, we recruited newly diagnosed patients with Parkinson’s disease with predominantly right-sided (*n* = 18), left-sided (*n* = 15) or symmetric nigrostriatal denervation (*n* = 15) assessed on dopamine PET. Detailed examinations of lateralized non-motor function included lacrimation, hand skin wrinkling, salivation, olfaction and pupillary function. Healthy controls were recruited for comparison. We observed a moderate-to-strong correlation between right–left asymmetry of putamen dopamine binding and asymmetry in pupillary redilation speed [Spearman’s rank correlation coefficient (*r_s_*) = −0.53, 95% confidence interval (−0.77; −0.14), *P* = 0.0084]. We also observed moderate correlations between non-negative putaminal asymmetry and lacrimation [*r_s_* = 0.35, (−0.00; 0.62), *P* = 0.0464] and word recognition [*r_s_* = 0.36, (0.01; 0.63), *P* = 0.0410]. However, none were significant after false discovery rate correction. We observed significant group differences in non-negative asymmetry in salivation (*P* = 0.0390, ANOVA) and a trend towards greater asymmetric lacrimation in participants with asymmetric striatal dopamine loss compared with healthy controls (*P* = 0.0330, unadjusted). Additionally, participants with asymmetric striatal dopaminergic binding showed greater, though non-significant, asymmetry in all pupillary measures compared with those with symmetric dopaminergic binding. In conclusion, this study contributes to our understanding of neurodegeneration progression in Parkinson’s disease and suggests a link between dopaminergic degeneration and non-motor measures related to autonomic function, particularly salivation, lacrimation and pupillary function. While our findings do not support a strict right–left hemispheric association between non-motor functions and dopaminergic degeneration, potential relationships may exist between these features and asymmetrical degeneration in other neuronal systems, such as the cholinergic.

## Teaser

Hansen *et al*. identify a link between asymmetric dopaminergic degeneration and asymmetry in autonomic function, particularly salivation, lacrimation and pupillary function. However, the study does not support a strict right–left hemispheric association between striatal dopamine asymmetry and non-motor functions involving other neurotransmitter systems.

## Graphical Abstract

**Graphical Abstract:**
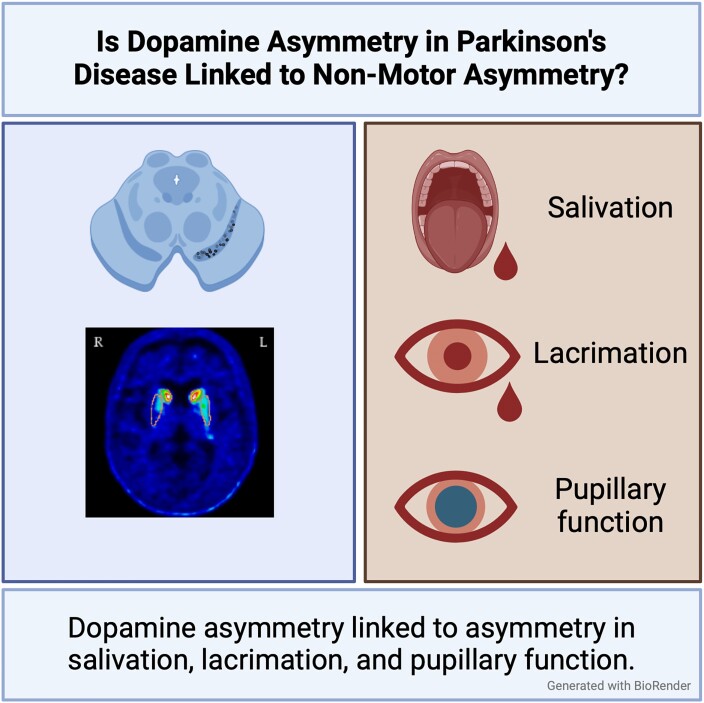

## Introduction

Asymmetry in motor function is a well-documented feature of Parkinson’s disease which correlates with asymmetry in striatal dopaminergic denervation.^1^ This asymmetry in dopaminergic integrity may be determined by asymmetric distribution of α-synuclein, the pathological hallmark of Lewy body disorders.^2,3^ As such, studying asymmetry in neurodegenerative disease offers a road to understanding how pathological processes develop and propagate between the cerebral hemispheres and throughout the nervous system.^4,5^

Assuming a prion-like propagation of α-synuclein aggregates from neuron to neuron, it is proposed that the hemisphere most affected by pathology is where it initially arises.^6^ Furthermore, given the broad impact of Lewy pathology on various neurotransmitter systems beyond the dopaminergic, it is theorized that other neuronal systems within the predominantly affected hemisphere are similarly affected.^7,8^ According to this hypothesis, asymmetry in dopaminergic degeneration would be expected to coincide with asymmetry in other neurotransmitter systems. Consequently, asymmetry in functions primarily dependent on dopaminergic integrity, such as motor function, should correlate with asymmetry in bilateral non-motor functions that rely on other cerebral systems, such as pupillary function. In support, a few studies have demonstrated that motor asymmetry may be associated with asymmetry in lateralized non-motor functions such as olfaction,^9,10^ cognition^11,12^ and skin wrinkling.^13^

However, prior research often concentrated on a restricted set of non-motor characteristics,^9,13-17^ employed a limited sample size^9,16,17^ and linked non-motor asymmetry to clinical motor asymmetry instead of utilizing an imaging-based reference point,^13,14,18,19^ which provides a more objective measure of neuronal degeneration. Overall, previous studies investigating the association between motor and non-motor asymmetry suggest that such links exist across various functions. However, there is considerable contradictory evidence, and many correlations have not been consistently replicated across different research groups and samples. Moreover, there is limited knowledge about motor asymmetry and potentially lateralized autonomic integrity, as evaluated through pupillary function, lacrimation and salivary gland secretion. Consequently, there is a demand for studies founded on prospectively collected data that assess asymmetry in non-motor parameters in patients selected through dopaminergic imaging.

Thus, our objective was to prospectively recruit newly diagnosed patients with Parkinson’s disease with symmetric and asymmetric dopaminergic degeneration for detailed clinical assessment. We hypothesized that right–left asymmetry in lateralized non-motor measures would correlate with right–left asymmetry in dopaminergic striatal integrity. We also hypothesized that patients with asymmetrical dopaminergic striatal degeneration would display more asymmetry on lateralized non-motor measures compared with patients with symmetric dopaminergic degeneration and healthy controls.

## Materials and methods

### Setting, design and participants

**Figure 1:**
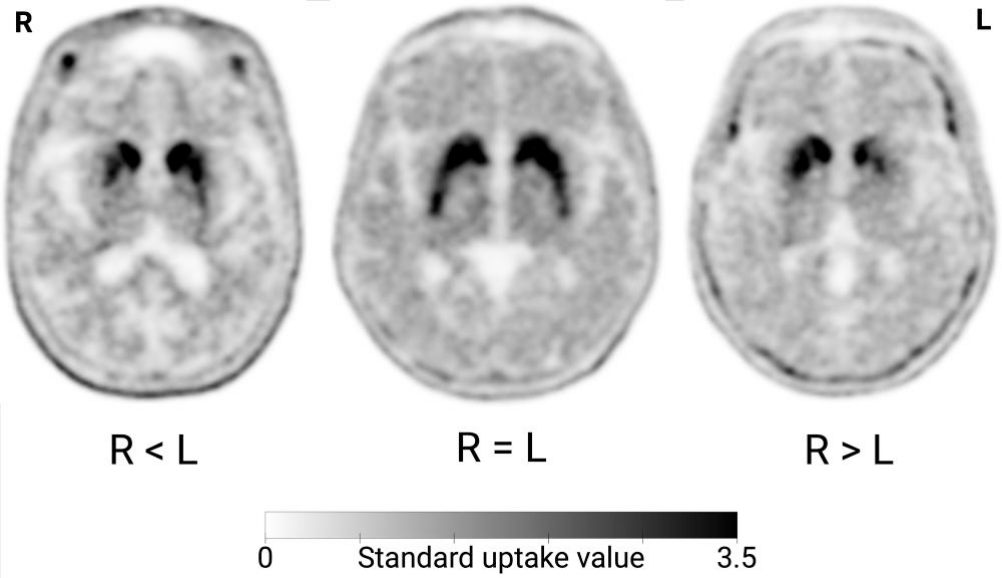
**Axial ^18^F-FE-PE2I PET images of 3 female participants of similar age with Parkinson’s disease, representing the 3 main groups.** Lower right compared with left putaminal binding (*left*), symmetric binding (*middle*) and lower left compared with right putamen binding (*right*).

This comparative cross-sectional study was carried out at the Department of Nuclear Medicine and PET, Aarhus University Hospital, Denmark, between September 2021 and July 2022. Based on consecutive ^18^F-fluoroethyl-(1R)-2β-carbomethoxy-3β-(4-iodophenyl)nortropane (^18^F-FE-PE2I) PET scans performed in-house, we prospectively recruited newly diagnosed subjects with Parkinson’s disease and (i) more right compared with left putaminal degeneration, (ii) more left compared with right putaminal degeneration and (iii) symmetric putaminal degeneration. We also recruited a group of healthy elderly controls matched for age and sex to the patient groups. Figure 1 illustrates the main comparison groups. Sample size was estimated to at least *n* = 15 in each group based on the results from 2 previous studies investigating olfaction and skin wrinkling in asymmetric patients with Parkinson’s disease.^9,13^ Detailed information about the recruitment is given in Supplementary Fig. 1. The inclusion criteria encompassed an unequivocally pathological PE2I PET scan with age-corrected unilateral or bilateral putaminal PE2I binding reduced more than 2 SDs below the healthy control mean. Symmetric patients were defined as those where the right and left putamen PE2I *z*-score differed by <0.5. In asymmetric patients, the right and left putamen PE2I *z*-score differed by 1.5 or more. From those meeting these criteria, we prioritized inviting the most recently investigated patients. The inclusion criteria also encompassed self-identifying as right-handed and a diagnosis of Parkinson’s disease <3 years old, as confirmed by a neurologist according to current diagnostic criteria.^20^ Case ascertainment was performed by telephone interview. General exclusion criteria were major psychiatric disorder, structural brain disease, current drug abuse or use of medication that mimics or affects sympathetic or parasympathetic function. Specific criteria for excluding data from the stimulated skin wrinkling assessment encompassed diabetes, peripheral neuropathy, previous surgery or radiation therapy to the upper extremities and major dermatological hand disease. Specific criteria for excluding data from olfactory tests encompassed current upper respiratory tract infection, nasal polyposis, allergic rhinitis and trauma or surgery to the nose. Controls were recruited through newspaper advertisement. Participants were invited for a 1-day visit during which clinical assessment of both motor and non-motor functions was conducted. Participants were asked to not consume coffee or tea within 12 h prior to the examinations or food within 6 h. All clinical examinations were performed prospectively by 1 examiner blinded to PE2I PET status. The study was approved by the local ethics committee and conducted according to the Declaration of Helsinki. All participants provided written informed consent.

### Clinical ^18^F-PE2I PET/CT imaging

Image acquisition and processing was performed as previously described^21^: a 10-min ^18^F-FE-PE2I PET was acquired using list mode on a Siemens Biograph Vision PET/CT camera. This acquisition commenced precisely 30 min following intravenous injection of ∼200 MBq ^18^F-FE-PE2I, with a low-dose CT scan performed immediately beforehand to enable attenuation correction and anatomical standardization. Image reconstruction utilized an iterative algorithm incorporating resolution recovery with 8 iterations, 5 subsets and an all-pass filter. All PE2I PET/CT scans underwent comprehensive qualitative and semi-quantitative analysis by a physician specialized in nuclear medicine with extensive expertise in interpreting dopamine scans. Initially, each scan underwent visual assessment for neurodegenerative patterns, including asymmetrical loss and/or predominant posterior putaminal reduction in striatal binding. To eliminate structural causes of reduced striatal binding, such as lacunar strokes and dilated perivascular spaces, a co-registered CT scan was employed and MRI when available. Subsequently, semi-quantitative analysis followed the method outlined by Marner *et al*.^22^ An automated segmentation algorithm delineated volumes of interest on static PE2I PET/CT images. Volumes of interest were defined for the left and right caudate nucleus and putamen, with the cerebellum grey matter serving as reference region. CT images were transformed to an MNI template, and segmentations were obtained by aligning the PET image with the CT image and applying the same transformation to the PET image. Average count values from the volumes of interest were extracted after reversing the PET image to its native space. Specific binding ratios for the left and right putamen and caudate nuclei were computed by dividing the volume of interest by the reference region minus 1. Additionally, putamen/caudate ratios were calculated separately for each hemisphere. Given the age-related decline in dopamine transporter density, binding values were adjusted for both patient groups using age-dependent decline estimates from in-house healthy control data. Subsequently, putamen and caudate *z*-scores were calculated by comparing patient specific binding ratios to an in-house dataset of 34 healthy, aged controls who underwent PE2I PET with identical methodology. A scan was considered pathological if both qualitative assessment and semi-quantitative values indicated clear abnormalities consistent with a neurodegenerative Lewy body disorder, in the absence of competing causes of decreased PE2I binding such as lacunar infarcts.

### Olfaction

Mono-rhinal odour identification was quantified using the blue version of the 16-item Sniffin’ Sticks test.^23^ First, participants performed the test using either the right or left nostril, with the contralateral nostril occluded using the index finger. After about 20 min, the same test was repeated using the contralateral nostril. The nostril sequence was randomly selected to diminish the risk of bias due to learning effects. Of note, we did not observe any learning effects in our data.

### Stimulated skin wrinkling

Following a baseline photo of the palmar surface of the hands, we applied 1 ml of topical analgesic crème to the palmar tip of the second, third, fourth and fifth finger on each hand.^13,24,25^ The crème contained 2.5% of lidocaine and 2.5% prilocaine. Then, the fingertips were covered with Tegaderm plaster. After 30 min, we removed the plasters, cleaned the fingertips using a dry cloth and obtained post-procedure photos. All photos were assessed by 1 examiner blinded to group status. The examiner rated wrinkles for each finger using a 5-point scale from 0 (wrinkling absent) to 4 (wrinkling completely distorts the pulp of the fingertip).^25^ Prior to the study, we evaluated feasibility, effectiveness and test–retest reliability of this method in 10 healthy controls (data not shown). For each finger, we calculated the difference between the post-procedure and pre-procedure score. We chose this method for its feasibility, as it has been shown to produce similar results to the water immersion method.^24^

### Stimulated salivary gland secretion

We placed an elongated cylindrical cotton roll in the gingivobuccal sulcus on each side of the upper mouth, opposite the second molar, thereby blocking the opening of the parotid duct. Subsequently, we stimulated saliva secretion by administering 0.5 ml of a 5% citric acid solution on the tongue every 30 s for 5 min. Saliva secretion was calculated by weighing the cotton pads before and after the experiment and calculating the difference in weight in milligrams.

### Pupillometry

We utilized pupillometry to measure pupil responses, as these are regulated by both the parasympathetic and sympathetic nervous systems. This approach allows us to distinguish and examine the contributions of each autonomic branch to pupil dynamics. After 5 min in a darkened examination room, we conducted mono-ocular pupillometry using the NeurOptics NPi-200 Pupillometer System on the right eye.^26^ After an additional 5 min, we conducted pupillometry on the left eye. The assessment covered baseline pupil diameter, minimum diameter post-flash, change from baseline, average constriction velocity, maximum constriction velocity, latency and average redilation speed. Visual acuity was measured in all patients using Snellen’s chart.

### Lacrimation

Lacrimation was quantified using Schirmer’s test,^27^ which measures the distance in millimetres that liquid has travelled on a filter paper. As such, the test is a simple, but rather coarse measure that can be challenging to interpret with precision.

### Blood pressure

Blood pressure was measured after 15 min of supine rest and then after 1, 2 and 3 min in the orthostatic position.^28^

### Motor function

We assessed motor function utilizing the Movement Disorder Society—Unified Parkinson’s Disease Rating Scale part III (UPDRS-III).^29^ Extremity scores for right and left sides were calculated from lateralized subscores. Motor speed was assessed with the alternate tapping test, in which participants use their index finger to alternately tap 2 stickers placed 20 cm apart.^30^ Patients were tested ON dopaminergic medication.

### Cognitive assessment

As a proxy measure of left-hemisphere function, we used the Boston naming test and a test of verbal memory from the Scales for Outcomes in Parkinson’s Disease-COGnition test.^31,32^ To ascertain right-hemisphere cognitive integrity, we used the Judgement of Line Orientation and measured object recognition from the Visual Object and Space Perception Battery.^33,34^ All tests were administered by 1 examiner supervised by an experienced neuropsychologist.

### Questionnaires

Non-lateralized non-motor symptoms were assessed utilizing the Non-Motor Symptoms Scale for Parkinson’s disease.^35^ Symptoms of REM sleep behaviour disorder was assessed with the REM Sleep Behaviour Disorder Screening Questionnaire.^36^

### Correlational analyses

We correlated asymmetry in putamen PE2I binding with asymmetry in bilateral motor and non-motor measures. First, we conducted these analyses among participants with asymmetrical putamen dopamine binding, quantifying asymmetry as the absolute difference between right and left measurements. These analyses were used to evaluate our first hypothesis that right–left asymmetry in lateralized non-motor measures would correlate with right–left asymmetry in dopaminergic striatal integrity. Next, as sensitivity analyses, we repeated the correlational analyses using a different measure of asymmetry, often referred to as an asymmetry index, calculated as the difference between right and left divided by the sum of right and left. Additionally, we repeated the correlational analyses using caudate PE2I binding instead of putamen binding and included participants with symmetrical putamen PE2I binding. Then, we correlated the non-negative value of right minus left putamen binding with non-lateralized non-motor variables, specifically the REM Sleep Behaviour Disorder Screening Questionnaire, Non-motor Symptom assessment Scale for Parkinson’s disease and maximum orthostatic systolic and diastolic blood pressure drop.

### Group comparisons

We compared the non-negative value of asymmetry in bilateral motor and non-motor measures, among participants with symmetric and asymmetric putaminal dopamine denervation and healthy controls. These analyses were used to evaluate our second hypothesis that patients with asymmetrical dopaminergic striatal degeneration would display more asymmetry on lateralized non-motor measures compared with patients with symmetric dopaminergic degeneration and healthy controls. We also compared right and left sides for bilateral motor and non-motor measures within groups and compared non-lateralized measures and demographic variables between groups.

### Statistical analysis

We used parametric tests for data with a normal distribution and non-parametric tests for non-normally distributed data. Non-parametric ANOVA and multiple-group comparisons were conducted using the Kruskal–Wallis test and corrected using Dunn’s multiple comparison procedure. Statistical significance was defined at *P* < 0.05. *P*-values from multiple correlations were analysed with the 2-stage step-up false discovery rate approach of Benjamini *et al*.^37^ at *Q* = 1%. Raw data were collected and stored using REDCap electronic data capture tools hosted at Aarhus University.^38^ Missing clinical observations are detailed in the tables and excluded from the analyses. We used STATA 17 for data administration and calculation of derived variables and GraphPad Prism 10 for analyses and data visualization.

## Results

We recruited 18 participants with lower right compared with left putaminal PE2I binding, 15 participants with lower left compared with right putaminal PE2I binding, 15 participants with symmetric putaminal PE2I binding and 16 elderly controls without symptoms or signs of neurodegenerative disease. The clinical and demographic characteristics of the participants are displayed in Table 1. The groups were similar overall; however, participants with symmetric putaminal PE2I binding showed higher age, greater maximum systolic drop, higher UPDRS-III motor scores and a trend towards higher scores on the REM Sleep Behaviour Disorder Screening Questionnaire compared with those with asymmetric binding. Also, participants with lower right-sided putaminal PE2I binding had longer time between PET scan and clinical investigations compared with participants with lower left-sided putaminal binding. Information on striatal PE2I binding is presented in Table 2, results from lateralized motor and non-motor assessments in Table 3 and pupillary measurements in Table 4. Extended versions of these tables including group comparisons are available in the Supplementary material. All participants self-identified as right-handed.

**Table 1:** Demographic and non-lateralized clinical information

	R < L *n* = 18	R = L *n* = 15	R > L *n* = 15	HC *n* = 16
Side of lowest putamen binding	Right (100%)	–	Left (100%)	16
Sex, male	13 (72%)	10 (67%)	7 (47%)	13 (81%)
Age, years	70 (61–74)	74 (71–79)	67 (58–73)	70 (69–73)
Alcohol, units/week	5 (2–7)	4 (2–7)	3 (2–7)	5 (3–14)
Smoking, pack years	6 ± 12	7 ± 14	5 ± 11	6 ± 10
Education, years	16 ± 3	19 ± 8	16 ± 5	17 ± 3
Handedness, right	18 (100%)	15 (100%)	15 (100%)	16 (100%)
LEDD, mg	200 (155–301)	200 (150–300)	104 (50–315)	–
H&Y, 0/I/II/III/IV	0/8/10/0/0	0/1/9/4/1	0/5/10/0/0	–
Duration of motor symptoms, years	4 (3–5)	4 (3–5)	2 (2–4)	–
Time since ^18^F-FE-PE2I PET, years	1.3 (0.3–1.6)	0.7 (0.4–1.0)	0.5 (0.5–0.7)	–
Orthostatic hypotension	6 (33%)	6 (40%)	4 (27%)	4 (25%)
Systolic blood pressure, mmHg	138 ± 17	150 ± 13	150 ± 22	144 ± 19
Diastolic blood pressure, mmHg	83 ± 12	83 ± 8	87 ± 15	80 ± 11
Supine hypertension ^a^	9 (50%)	12 (80%)	11 (73%)	9 (56%)
Maximum systolic drop, mmHg	−9 ± 21	−21 ± 17	−8 ± 17	−13 ± 9
Maximum diastolic drop, mmHg	−2 ± 13	−2 ± 9	0 ± 11	1 ± 5
RBDSQ, score	3 (2–5)	5 (3–6)	2 (2–5)	2 (2–4)
Probable RBD	3 (17%)	6 (40%)	3 (20%)	3 (19%)
NMSS, score	49 (33–56)	37 (29–47)	24 (12–43)	14 (7–19)
UPDRS-III, score	28 (16–35)	37 (26–41)	24 (23–33)	7 (5–12)

Basic demographic and non-lateralized clinical variables for 18 participants with lower right compared with left putaminal ^18^F-FE-PE2I PET binding (R < L), 15 participants with lower left compared with right putaminal ^18^F-FE-PE2I PET binding (R > L), 15 participants with symmetrical putaminal ^18^F-FE-PE2I PET binding (R = L) and 16 healthy elderly control subjects (HC). Continuous variables are reported as mean ± standard deviation if normally distributed and median (interquartile range) if non-normally distributed. Categorical variables are presented with frequency and percentage. An extended version of this table including group comparisons is available in the Supplementary material (Supplementary Table 1).

H&Y, Hoehn and Yahr scale; LEDD, levodopa equivalent daily dose; NMSS, Non-Motor Symptoms Scale for Parkinson’s Disease; RBD, REM sleep behavior disorder; RBDSQ, REM Sleep Behavior Disorder Screening Questionnaire; UPDRS-III, Movement Disorder Society—Unified Parkinson’s Disease Rating Scale part III.

^a^Defined as systolic blood pressure > 140 mmHg or diastolic blood pressure > 90 mmHg.

**Table 2:** Striatal ^18^F-FE-PE2I PET binding

	R < L *n* = 18	R = L *n* = 15	R > L *n* = 15
**Putamen, specific binding ratio**			
Right	1.70 (1.63–2.01)	1.62 (1.47–1.91)	3.02 (2.61–3.36)
Left	2.58 (2.40–2.97	1.54 (1.27–1.73)	1.93 (1.60–2.21)
Average	2.20 (2.02–2.39)	1.63 (1.42–1.79)	2.50 (2.06–2.71)
**Caudate, specific binding ratio**			
Right	4.09 (3.32–4.40)	3.19 (2.96–3.59)	4.06 (3.86–4.70)
Left	4.39 (3.74–4.72)	3.10 (2.91–3.42)	3.78 (3.53–4.46)
Average	4.25 (3.47–4.47)	3.10 (2.81–3.51)	3.99 (3.64–4.54)
**Putamen/caudate, specific binding ratio**			
Right	0.47 (0.41–0.57)	0.53 (0.47–0.59)	0.66 (0.61–0.73)
Left	0.63 (0.55–0.87)	0.51 (0.47–0.56)	0.47 (0.41–0.52)
Average	0.55 (0.50–0.72)	0.52 (0.46–0.57)	0.57 (0.53–0.63)

^18^F-FE-PE2I PET binding in the putamen and caudate of 18 participants with lower right compared with left putaminal ^18^F-FE-PE2I PET binding (R < L), 15 participants with lower left compared with right putaminal ^18^F-FE-PE2I PET binding (R > L) and 15 participants with symmetrical putaminal ^18^F-FE-PE2I PET binding (R = L). All variables are continuous and non-normally distributed and reported as median (interquartile range). An extended version of this table including group comparisons is available in the Supplementary material (Supplementary Table 2).

**Table 3:** Lateralized motor and non-motor assessment

	R < L *n* = 18	R = L *n* = 15	R > L *n* = 15	HC *n* = 16
**Cognition**				
Word recall^a^	6 (5–7)	6 (3–7)	7 (5–9)	8 (7–10)
Word recognition^a^	10 (9–10)	10 (9–10)	10 (10–10)	10 (10–10)
Line orientation^b^	24 (22–28)^d^	23 (18–26)	26 (22–29)	27 (25–28)
Line bisection^c^, mm	2 (−20–33)	43 (17–64)	10 (−18–28)	−2 (−16–38)
Line bisection^e^, absolute	22 (9–44)	44 (26–64)	19 (10–28)	21 (11–43)
**Olfaction, score**				
Right nostril	6 (5–9)^d^	6 (4–9)	7 (5–8)^f^	11 (10–13)
Left nostril	6 (5–9)^d^	6 (5–7)	7 (5–8)^f^	12 (9–13)
Average	6 (5–8)^d^	6 (5–8)	7 (5–8)^f^	11 (10–13)
**Skin wrinkling, score**				
Right hand	1.6 (1.0–3.0)^g^	1.8 (1.3–2.3)^g^	2.0 (1.5–2.8)	1.6 (1.3–3.3)
Left hand	1.3 (1.0–2.0)^g^	1.8 (1.5–2.3)^g^	2.0 (1.8–3.5)	2.3 (1.3–2.5)
Average	1.4 (1.0–2.6)^g^	1.9 (1.4–2.1)^g^	2.3 (1.9–3.0)	2.0 (1.3–2.9)
**Salivation, mg**				
Right cheek	1.89 (1.58–2.07)^d^	2.06 (1.92–2.17)	2.11 (1.98–2.16)	2.07 (2.01–2.17)
Left cheek	2.11 (1.94–2.24)^d^	2.06 (1.52–2.20)	2.08 (1.88–2.11)	2.04 (1.88–2.11)
Average	1.98 (1.53–2.08)^d^	2.05 (1.89–2.19)	2.05 (1.89–2.14)	2.06 (1.94–2.11)
**Lacrimation, mm**				
Right eye	5 (4–9)	5 (4–8)	8 (5–14)	8 (5–16)
Left eye	5 (3–8)	5 (3–6)	5 (4–10)	7 (2–15)
Average	6 (4–8)	5 (4–7)	8 (4–11)	8 (3–14)
**Alternate tapping test, taps/min**				
Right	149 (125–158)	129 (112–147)	149 (120–172)	166 (148–174)
Left	132 (119–150)	119 (109–124)	155 (134–170)	154 (146–168)
Average	143 (122–154)	122 (110–134)	156 (115–174)	160 (149–170)
**UPDRS-III, extremity score**				
Right	7 (2–10)	11 (8–16)	14 (9–17)	3 (2–5)
Left	14 (8–19)	12 (10–17)	6 (3–9)	3 (2–6)
Average	10 (6–14)	12 (11–14)	9 (8–13)	3 (2–5)

Lateralized motor and non-motor assessments in 18 participants with lower right compared with left putaminal ^18^F-FE-PE2I PET binding (R < L), 15 participants with lower left compared with right putaminal ^18^F-FE-PE2I PET binding (R > L), 15 participants with symmetrical putaminal ^18^F-FE-PE2I PET binding (R = L), a combined group of participants with asymmetrical putaminal ^18^F-FE-PE2I PET binding (R ≠ L) and 17 healthy elderly control subjects (HC). All variables are continuous and reported as median (interquartile range). An extended version of this table including group comparisons is available in the Supplementary material (Supplementary Table 3).

^a^Assessed with the SCOPA-COG (Scales for Outcomes in Parkinson’s Disease-COGnition).

^b^Correct answers on the Benton Judgement of Line Orientation test.

^c^Overshoot in mm to the right on the Line bisection test.

^d^Missing data for 1 patient.

^e^Absolute overshoot in mm.

^f^Missing data for 2 patients.

^g^Data from 2 patients excluded.

**Table 4:** Pupillary measurements

	R < L *n* = 18	R = L *n* = 15	R > L *n* = 15	HC *n* = 16
**Baseline diameter, mm**				
Right	5.3 ± 1.0	4.9 ± 1.0	5.6 ± 1.1	4.4 ± 1.1
Left	5.0 ± 0.8	4.9 ± 0.9	5.4 ± 1.0	4.3 ± 1.1
Average	5.1 ± 0.6	4.9 ± 0.9	5.5 ± 1.0	4.4 ± 1.1
**Minimum diameter post-flash, mm**				
Right	3.2 ± 0.6	3.0 ± 0.7	3.3 ± 0.8	2.6 ± 0.6
Left	3.0 ± 0.6	2.9 ± 0.6	3.3 ± 0.8	2.6 ± 0.7
Average	3.1 ± 0.6	3.0 ± 0.6	3.3 ± 0.8	2.6 ± 0.7
**Change from baseline, %**				
Right	39 ± 5	40 ± 6	41 ± 7	43 ± 5
Left	41 ± 3	39 ± 6	39 ± 7	41 ± 5
Average	40 ± 3	40 ± 6	40 ± 6	42 ± 3
**Average constriction velocity, mm/s**				
Right	2.5 ± 0.6	2.7 ± 0.5	2.7 ± 0.5	2.5 ± 0.6
Left	2.5 ± 0.3	2.5 ± 0.5	2.5 ± 0.5	2.3 ± 0.6
Average	2.5 ± 0.4	2.6 ± 0.5	2.6 ± 0.5	2.4 ± 0.5
**Maximum constriction velocity, mm/s**				
Right	4.2 ± 0.9	4.3 ± 0.8	4.4 ± 0.9	4.3 ± 0.9
Left	4.2 ± 0.5	4.3 ± 0.8	4.2 ± 0.9	4.1 ± 0.9
Average	4.2 ± 0.6	4.3 ± 0.7	4.3 ± 0.8	4.1 ± 0.7
**Latency, s**				
Right	0.2 ± 0.0	0.2 ± 0.0	0.2 ± 0.0	0.2 ± 0.0
Left	0.2 ± 0.0	0.2 ± 0.0	0.2 ± 0.0	0.2 ± 0.0
Average	0.2 ± 0.0	0.2 ± 0.2	0.2 ± 0.0	0.2 ± 0.0
**Average redilation speed, mm/s**				
Right	1.0 ± 0.2	0.9 ± 0.2	1.1 ± 0.2	1.0 ± 0.3
Left	0.8 ± 0.2	0.8 ± 0.2	1.1 ± 0.4	0.8 ± 0.2
Average	0.9 ± 0.2	0.9 ± 0.2	1.1 ± 0.3	0.8 ± 0.2

Pupillary measurements in right and left eye in 18 participants with lower right compared with left putaminal ^18^F-FE-PE2I PET binding (R < L), 15 participants with lower left compared with right putaminal ^18^F-FE-PE2I PET binding (R > L), 15 participants with symmetrical putaminal ^18^F-FE-PE2I PET binding (R = L) and 17 healthy elderly control subjects (HC). All variables are continuous and reported as mean ± standard deviation. An extended version of this table including group comparisons is available in the Supplementary material (Supplementary Table 4).

### Group comparisons of non-negative values of asymmetry

**Figure 2:**
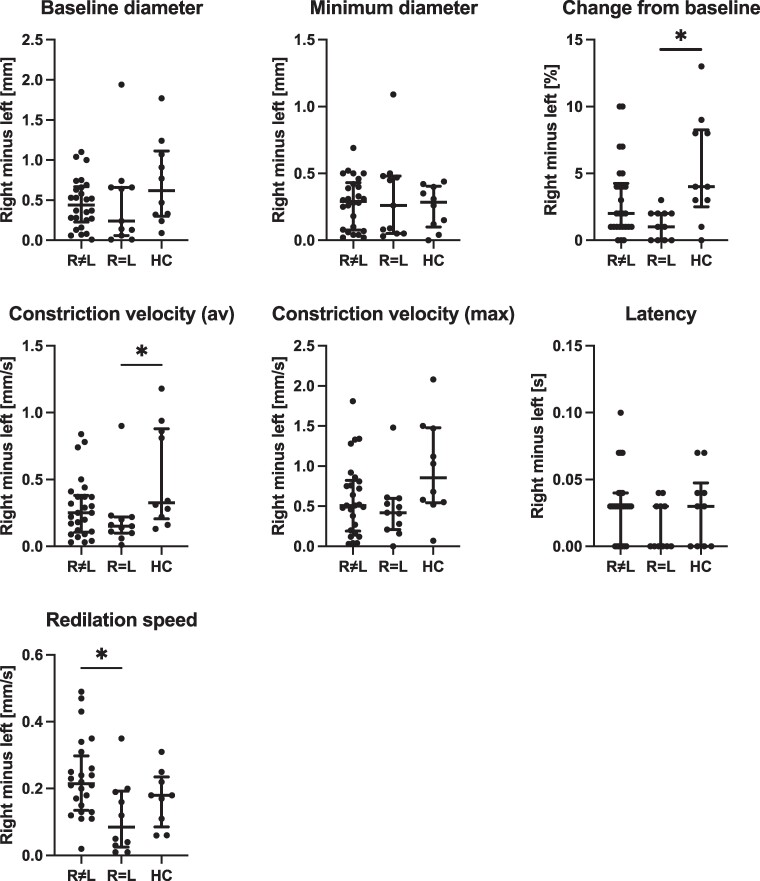
**Pupil asymmetry in subjects with asymmetrical versus symmetrical striatal PE2I binding and healthy controls. Individual data points represent pupil asymmetry for each subject, calculated as the difference between the right and the left eye.** The panels compare asymmetry in specific measures of pupillary function across 3 groups: subjects with asymmetric striatal dopamine binding (R≠L, *n* = 33), subjects with symmetric binding (R = L, *n* = 15) and age-matched healthy controls (HC, *n* = 16). Group differences were analysed using the Kruskal–Wallis test with Dunn’s *post hoc* correction. Statistical significance (*P* < 0.05) after Dunn’s correction is indicated by an asterisk. av, average; max, maximum.

We detected a significant difference in non-negative asymmetry regarding stimulated salivation among the groups, which remained consistent whether we quantified asymmetry as the absolute difference between left and right sides (*P* = 0.0390) or as an index (*P* = 0.0403). This distinction was primarily driven by heightened salivary asymmetry observed in participants with asymmetric striatal PE2I binding compared with healthy controls. We also noted a difference among the groups in non-negative asymmetry concerning average pupil redilation speed (*P* = 0.0243). This difference was primarily due to heightened asymmetry observed in participants with asymmetric compared with symmetrical striatal PE2I binding (*P* = 0.0213). Furthermore, we observed a difference among the groups in non-negative asymmetry concerning pupil change from baseline (*P* = 0.0168) and average pupil constriction velocity (*P* = 0.0325) (Fig. 2). These differences were primarily driven by greater symmetry observed in participants with symmetrical striatal PE2I binding compared with healthy controls (*P* = 0.0132 and *P* = 0.0257, respectively). We also observed a tendency towards greater asymmetrical lacrimation among participants with asymmetric striatal PE2I binding compared with healthy controls, with a significance level of *P* = 0.0330 (unadjusted) and *P* = 0.0857 (adjusted). However, we found no group differences in non-negative asymmetry for alternate tapping, odour identification, stimulated skin wrinkling or pupil measurements of baseline diameter, minimum diameter post-flash, maximum constriction velocity or latency. Notably, for 7 out of 7 (100%) of non-negative measures of pupil asymmetry defined as the difference between right minus left (Fig. 2) and for 6 out of 7 (86%) when defined as an index, participants with asymmetric striatal PE2I binding exhibited greater absolute asymmetry compared with participants with symmetrical striatal PE2I binding.

There was a significant difference in non-negative right minus left asymmetry observed in extremity UPDRS-III scores among participants with asymmetric striatal PE2I binding, those with symmetric ^18^F-FE-PE2I binding and healthy controls (ANOVA conducted using Kruskal–Wallis test, *P* < 0.0001). After conducting Dunn’s multiple comparison test, we found that non-negative asymmetry in extremity UPDRS-III scores was significantly higher in asymmetric participants compared with both symmetric participants (*P* = 0.0038) and healthy controls (*P* < 0.0001). However, there was no significant difference in UPDRS-III asymmetry between symmetric patients and healthy controls. Similar results were found when using the asymmetry index.

### Correlations between asymmetry in striatal dopamine binding and asymmetry in clinical measures

**Figure 3:**
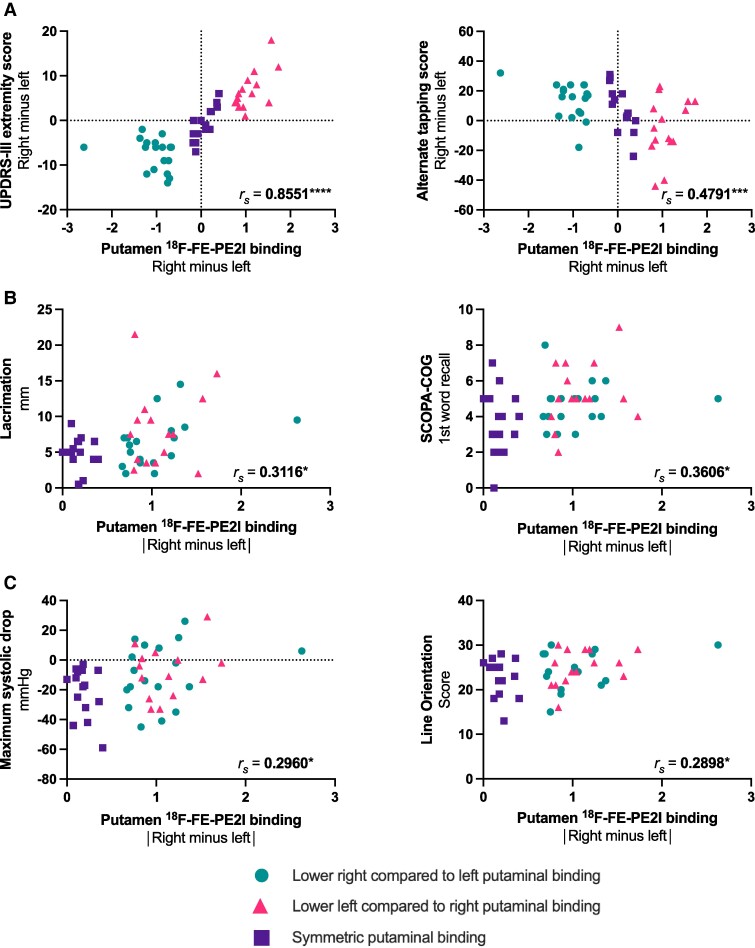
**Spearman’s rank correlations between asymmetry in putamen ^18^F-FE-PE2I specific binding ratio and motor and non-motor measures in patients with Parkinson’s disease.** (**A**) Correlations between right–left asymmetry of putamen ^18^F-FE-PE2I PET binding and right–left asymmetry in motor measures among all participants with Parkinson’s disease. (**B**and **C**) Correlations between non-negative right minus left putamen ^18^F-FE-PE2I PET binding and selected non-motor measures. Circles mark subjects with lower right compared with left putaminal binding (*n* = 18), triangles mark subjects with lower left compared with right putaminal binding (*n* = 15), and squares mark participants with symmetric binding (*n* = 15). * *P* < 0.05, ** *P* < 0.01, *** *P* < 0.001, **** *P* < 0.0001. SCOPA-COG, Scales for Outcomes in Parkinson’s Disease-COGnition; UPDRS-III, Movement Disorder Society—Unified Parkinson’s Disease Rating Scale part III.

Among asymmetric participants, we observed a strong correlation between right–left asymmetry of putamen PE2I binding and asymmetry in UPDRS-III extremity score [Spearman’s rank correlation coefficient (*r_s_*) = 0.73, 95% confidence interval (0.50; 0.86), *P* < 0.0001], alternate tapping [*r_s_* = −0.45 (−0.69; −0.12), *P* = 0.0082] and average pupillary redilation speed [*r_s_* = −0.53 (−0.77; −0.14), *P* = 0.0084]. However, after false discovery rate correction, only the correlation with UPDRS-III extremity score was significant, and we found no significant correlations with asymmetry in other bilateral non-motor functions. When using the asymmetry index, the correlations between lateralized putaminal PE2I binding and motor function remained, but the correlation with average pupillary redilation speed disappeared. Also, only the correlation with UPDRS-III extremity score was significant after false discovery rate correction, and no correlations with other lateralized non-motor tests emerged. Similar results were found when re-running the analyses in a combined group of all participants with Parkinson’s disease, except here the correlations between right–left asymmetry in putamen PE2I binding and right–left asymmetry in both UPDRS-III extremity score and alternate tapping remained after correcting the *P*-values (Fig. 3A). Overall, similar results were found when using caudate right–left asymmetry or asymmetry index in all participants with Parkinson’s disease or restricted to those with asymmetrical striatal binding.

Moreover, among asymmetric participants, we observed a correlation between non-negative right minus left putamen PE2I binding and lacrimation [*r_s_* = 0.35 (−0.00; 0.62), *P* = 0.0464], word recognition [*r_s_* = 0.36 (0.01; 0.63), *P* = 0.0410], average putamen PE2I binding [*r_s_* = 0.51 (0.19; 0.73), *P* = 0.0026] and alternate tapping score [*r_s_* = 0.51 (0.19; 0.73), *P* = 0.0024]. The correlations between non-negative right minus left putamen binding and lacrimation, word recognition, average putamen PE2I binding and alternate tapping score remained when re-running the correlations in a combined group of all participants with Parkinson’s disease (Fig. 3B). Additionally, correlations between non-negative right minus left putamen binding and maximum systolic drop and line orientation emerged (Fig. 3C). However, none were significant after false discovery rate correction. All correlations are presented in Supplementary Tables 5–14.

## Discussion

This study investigated whether asymmetry in dopaminergic degeneration correlated with asymmetry in functions that do not primarily rely on dopaminergic integrity, specifically pupillary function, lacrimation, salivation, olfaction, lacrimation, skin wrinkling and certain cognitive measures. Documenting such as correlation would support the idea that Lewy pathology asymmetrically affects both dopaminergic and non-dopaminergic systems and that degeneration across different neurotransmitter systems and their associated functions may be interconnected.

In this study, several findings and trends indicated that subjects with symmetric dopaminergic degeneration displayed more symmetry in some non-motor measures, including salivation, lacrimation and pupillary function, compared with patients with asymmetric dopaminergic degeneration. These findings support the overall hypothesis that patients with asymmetric dopaminergic striatal degeneration display more asymmetry also in some non-motor domains compared with patients with symmetric dopaminergic loss and healthy controls. Participants with symmetric dopamine reductions also showed greater maximum systolic drop and a trend towards higher scores on the REM Sleep Behaviour Disorder Questionnaire compared with those with asymmetric loss. This finding corroborates previous reports that symmetric dopamine degeneration, orthostatic hypotension and REM Sleep Behaviour Disorder cluster together, in support of a body-first subtype of Lewy body disease.^6,39,40^ Of note, the symmetric patient group was 5 years older than the asymmetric group, so age-related blood pressure dysregulation could also be a factor.

We observed a moderate-to-strong correlation between right–left asymmetry of putamen dopamine integrity and asymmetry in pupillary redilation speed and a weak-to-moderate correlation between non-negative right minus left putamen binding and lacrimation and word recognition. However, these findings were not significant after false discovery rate correction. Thus, our results did not support the hypothesis that right–left asymmetry in lateralized non-motor measures correlate with right–left asymmetry in dopaminergic striatal integrity.

The absence of these correlations may indicate that Lewy body disease is not characterized by underlying asymmetric distribution of Lewy pathology in brain-first cases, as proposed by the synuclein, origin and connectome model.^6^ However, it is also possible that many of the non-motor features examined here do not manifest with the same striking asymmetry as that seen in the motor system. In addition, some of the methodologies employed may have been suboptimal as suggested by the lack of even group-wise reductions in patient versus control comparisons.

Furthermore, while the relationships between right–left asymmetry in lateralized non-motor measures and asymmetry in striatal dopaminergic integrity were not significant, there could indeed be relationships between these features and asymmetrical degeneration of other neuronal systems, such as serotonergic or cholinergic systems.^8,41^

We found no correlations between asymmetry in stimulated skin wrinkling and striatal dopamine degeneration. Surprisingly, we also did not find significant group differences or even trends towards overall reduced stimulated skin wrinkling in patients with Parkinson’s disease compared with healthy controls. This was surprising, since patients with Parkinson’s disease undergo severe sympathetic degeneration and exhibit greater sympathetic dysfunction compared with healthy controls.^42^ Stimulated skin wrinkling on the fingertip has been suggested to represent the function of the sympathetic nervous system in that extremity.^25^ Until now, skin wrinkling has been studied in only 1 small cohort of patients with Parkinson’s disease.^13^ However, the absence of patient versus control differences in the present study suggests that stimulated skin wrinkling in our set-up was not a reliable method for assessing sympathetic skin integrity in Parkinson’s disease.

Previous studies have found that patients with Parkinson’s disease exhibit decreased lacrimation and salivation compared with healthy controls.^43-46^ Both functions rely on parasympathetic gland integrity. To our knowledge, asymmetry in lacrimation and salivation has not been previously studied in Parkinson’s disease. Here, we found that subjects with asymmetric putaminal dopamine degeneration displayed more asymmetry in salivation (statistically significant) and lacrimation (non-significant after adjustment) compared with controls. However, we found no indications of right–left asymmetric association between lacrimation or salivation and dopamine degeneration, nor any significant group differences in overall secretion amounts, except for a trend towards less salivation from the right cheek in the patient group with predominantly right-sided dopamine loss (Supplementary Table 3).

Several studies reported pupillary dysfunction in patients with Parkinson’s disease.^26,47,48^ The integrity of the autonomic innervation of the pupillary muscles is tested with a pupillometer, assessing both parasympathetic and sympathetic pathways. Previous studies were not designed to address asymmetry because they either administered different dilating drops in each eye, making comparison inappropriate, or calculated averages for both eyes. After correction, no right–left pupillary measures correlated with putamen dopamine integrity. However, we did observe differences in non-negative asymmetry among the groups in certain pupillary measures (Fig. 2). Notably, for nearly all measures of pupillary function, regardless of how asymmetry was defined, participants with asymmetric striatal PE2I binding showed greater absolute asymmetry compared with those with symmetrical striatal PE2I binding. This suggests that in Parkinson’s disease, symmetric pupillary dysfunction is linked to symmetrical dopamine degeneration. To our knowledge, this study is the first to evaluate pupillary asymmetry in relation to striatal dopamine integrity in Parkinson’s disease.

Hemihyposmia in Parkinson’s disease has been investigated in a few studies with conflicting results.^49^ Two studies did document reduced olfaction in the nostril contralateral to the body side most affected by motor symptoms.^9,16^ A case study supported these findings.^50^ However, our study, which correlated hemihyposmia with lateralized striatal dopamine integrity, found no significant correlations. The relationship between self-reported or measured hyposmia and the underlying aetiological causes is complex and not fully understood. The most used olfaction tests in the Parkinson’s field are forced-choice odour identification tests, such as the Sniffin’ Sticks identification test employed here or the University of Pennsylvania Smell Identification Test.^51^ Subjects need to correctly identify an odour among 4 choices. This task presumably requires relatively intact primary sensory functions, but also cognitive, memory and semantic functions. This may explain an intriguing observation seen in 2 independent post-mortem studies, namely that hyposmia based on odour identification shows no or minimal correlation with the amount of Lewy pathology in the olfactory bulb itself but stronger correlation with the total burden of Lewy pathology in the CNS.^52,53^ In short, hyposmia seems to be an indicator of global Lewy pathology more so than of olfactory bulb Lewy pathology. If this interpretation is correct, the complete absence of asymmetric hyposmia in our study may be less surprising. Future studies should investigate asymmetric hyposmia with chemosensory threshold tests, which does not require olfactory memory, but just the ability to detect whether an odour is present or not.^23^

Lateralized cognitive functions are also affected in Parkinson’s disease.^19,54,55^ However, in this study, we found no convincing correlations between striatal dopamine asymmetry and some cognitive functions presumed to be lateralized to the right or left hemisphere. This lack of association may be due to these functions being less lateralized than motor functions or inadequate sample size. Furthermore, additional pathologies beyond Lewy body disease may contribute to cognitive decline, potentially obscuring any associations.^56^

We observed an interesting difference between the UPDRS-III and alternating tapping data (Fig. 2). Whereas a very strong left–right association was seen between UPDRS-III scores and PE2I binding asymmetry, the association was not quite as strong for alternating tapping. This difference was probably caused by handedness, as shown by our right-handed healthy controls, who on average could tap 12 times more with the dominant hand in 1 min (Table 3). In short, a predominantly left-sided parkinsonism will lead to a widening of this dominant versus non-dominant gap, whereas right-sided parkinsonism first must close this physiological gap. Although not surprising, this observation needs to be considered when studying motor asymmetry with more advanced quantitative measures.^30^

Disease duration influences asymmetry in Parkinson’s disease. Over time, subjects tend to become increasingly symmetric when assessed using the asymmetry index of putaminal dopamine degeneration.^5,57,58^ In our study, we aimed to maximize the potential power of asymmetrical measures while balancing disease duration across all 3 groups by recruiting newly diagnosed subjects. However, we found that subjects with lower left compared with right putaminal binding had shorter duration of motor symptoms than those with lower right compared with left putaminal binding (medican 2 versus 4 years, *P* = 0.0980; see Supplementary Table 1). Additionally, the combined group of subjects with asymmetric putaminal binding had fewer years of motor symptoms compared with those with symmetric putaminal binding (3 versus 4 years, *P* = 0.6020). Since these group differences were not significant, we anticipate that they did not substantially affect our results.

The effective use of antiparkinsonian medication is another factor that may attenuate clinical asymmetry.^58^ Although there were group differences in levodopa equivalent daily dosages, these differences were not statistically significant. Collectively, future studies should aim to minimize both disease duration and antiparkinsonian medication usage.

This study has several limitations. First, subjects with symmetric dopamine degeneration were older than those with asymmetric dopamine degeneration (Supplementary Table 1). This unintended sample bias reflects a strong association between age and dopamine symmetry in the underlying population (data not shown). Future studies comparing symmetric and asymmetric patients with Parkinson’s disease should carefully match for age. Second, participants with lower right-sided putaminal PE2I binding had longer time between PET scan and clinical investigations compared with those with lower left-sided binding (Supplementary Table 1). This discrepancy likely reflects the higher prevalence of left-sided putaminal dopamine degeneration, necessitating a longer search to find enough subjects with right-sided degeneration for equally sized samples. Third, we focused on lateralized non-motor measures related to autonomic integrity, olfaction and cognition because these are well-established in Parkinson’s disease asymmetry and were practical to conduct in our setting. While less studied in relation to asymmetry, domains such as pain, touch and hearing may provide valuable insights for future research.^1,59,60^ Although our modest sample size was carefully considered and supported by power calculations, a larger sample may have provided greater statistical power to support our conclusions. The generalizability of this study’s results is limited due to the stringent inclusion criteria.

## Conclusion

This study holds importance for how we understand the inter-hemispheric progression of neurodegeneration in Parkinson’s disease. Our findings indicate that asymmetric dysfunction goes beyond dopamine-related motor symptoms to include autonomic functions such as salivation, lacrimation and pupillary response. However, the study does not support a strict right–left hemispheric association in non-motor systems, at least not related to olfaction, stimulated skin wrinkling and some cognitive tests. This lack of association may be due to these functions being less lateralized than motor functions or potentially suboptimal methodologies, as some measurements did not differentiate between Parkinson’s and control groups. Nonetheless, while our findings do not support a strict right–left hemispheric association between non-motor functions and dopaminergic degeneration, potential relationships may exist between these features and asymmetrical degeneration in other neuronal systems, such as the cholinergic.

## Supplementary Material

fcaf002_Supplementary_Data

## Data Availability

The data supporting the findings of this study are available on request from the corresponding author. Data sharing will require a formal data sharing agreement approved by the relevant local ethics committees.
