# Supplementary material for: Non-motor asymmetry and dopamine degeneration in Parkinson’s disease
Source: Brain Commun. 2025 Jan 6;7(1):fcaf002. doi: 10.1093/braincomms/fcaf002 (PMC11752486; doi:10.1093/braincomms/fcaf002)
Supplement: fcaf002_Supplementary_Data [file fcaf002_supplementary_data.docx]

# Supplementary Material


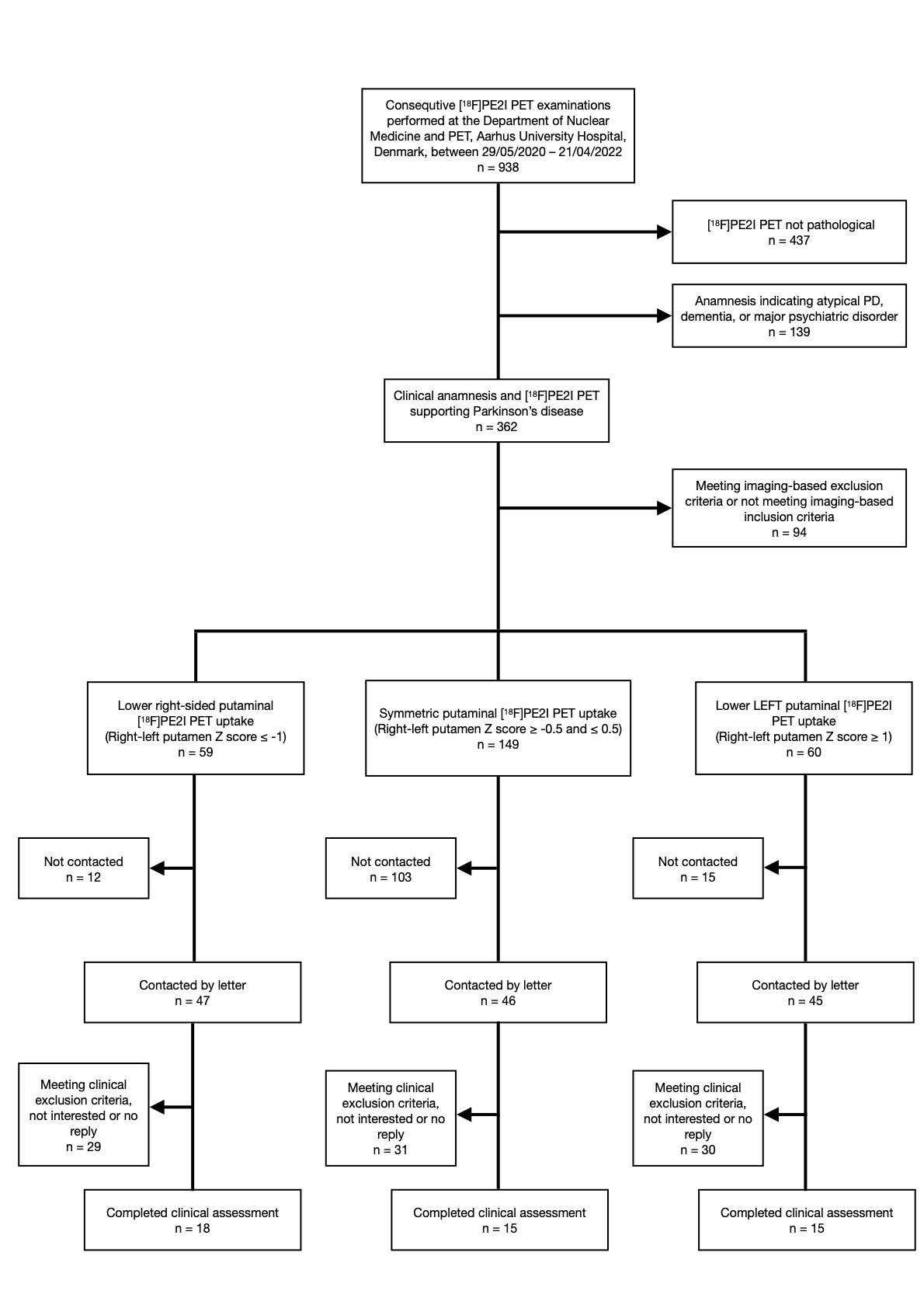


**Supplementary figure 1. Flow chart of participant recruitment process.**

| **Supplementary table 1. Demographic and non-lateralized clinical information** | | | | | |  |  |
| --- | --- | --- | --- | --- | --- | --- | --- |
|  | **R < L**  **n = 18** | **R = L**  **n = 15** | **R > L**  **n = 15** | **R ≠ L**  **n = 33** | **HC**  **n = 16** | **R = L**  **R ≠ L** | **R < L**  **R > L** |
| Sex, male | 13 (72%) | 10 (67%) | 7 (47%) | 20 (61%) | 13 (81%) | 0.7571 | 0.1686 |
| Age, years | 70 (61–74) | 74 (71–79) | 67 (58–73) | 69 (61–73) | 70 (69–73) | **0.0022** | 0.3615 |
| Alcohol, units/week | 5 (2–7) | 4 (2–7) | 3 (2–7) | 3 (2–7) | 5 (3–14) | 0.6300 | 0.5838 |
| Smoking, pack years | 6 ± 12 | 7 ± 14 | 5 ± 11 | 6 ± 11 | 6 ± 10 | 0.8017 | 0.3613 |
| Education, years | 16 ± 3 | 19 ± 8 | 16 ± 5 | 16 ± 4 | 17 ± 3 | 0.3110 | 0.8931 |
| Handedness, right | 18 (100%) | 15 (100%) | 15 (100%) | 33 (100%) | 16 (100%) | – | – |
| LEDD, mg | 200 (155–301) | 200 (150–300) | 104 (50–315) | 180 (100–303) | – | 0.6312 | 0.1179 |
| H&Y, 0/I/II/III/IV | 0/8/10/0/0 | 0/1/9/4/1 | 0/5/10/0/0 | 0/13/20/0/0 | – | – | – |
| Duration of motor symptoms, years | 4 (3–5) | 4 (3–5) | 2 (2–4) | 3 (2–5) | – | 0.6020 | 0.0980 |
| Time since ^18^F-FE-PE2I PET, years | 1.3 (0.3–1.6) | 0.7 (0.4–1.0) | 0.5 (0.5–0.7) | 0.7 (0.4 – 1.4) | – | 0.3020 | **0.0218** |
| Orthostatic hypotension | 6 (33%) | 6 (40%) | 4 (27%) | 10 (30%) | 4 (25%) | 0.5275 | 0.7220 |
| Systolic blood pressure, mmHg | 138 ± 17 | 150 ± 13 | 150 ± 22 | 143 ± 20 | 144 ± 19 | 0.2552 | 0.0921 |
| Diastolic blood pressure, mmHg | 83 ± 12 | 83 ± 8 | 87 ± 15 | 85 ± 13 | 80 ± 11 | 0.5345 | 0.3907 |
| Supine hypertension ^a^ | 9 (50%) | 12 (80%) | 11 (73%) | 20 (61%) | 9 (56%) | 0.3220 | 0.2844 |
| Maximum systolic drop, mmHg | -9 ± 21 | -21 ± 17 | -8 ± 17 | -9 ± 19 | -13 ± 9 | **0.0332** | 0.8420 |
| Maximum diastolic drop, mmHg | -2 ± 13 | -2 ± 9 | 0 ± 11 | -1 ± 12 | 1 ± 5 | 0.7506 | 0.5612 |
| RBDSQ, score | 3 (2–5) | 5 (3–6) | 2 (2–5) | 3 (2–5) | 2 (2–4) | 0.1198 | 0.6310 |
| Probable RBD | 3 (17%) | 6 (40%) | 3 (20%) | 6 (18%) | 3 (19%) | 0.1524 | >0.9999 |
| NMSS, score | 49 (33–56) | 37 (29–47) | 24 (12–43) | 37 (17–53) | 14 (7–19) | 0.7623 | 0.0660 |
| UPDRS-III, score | 28 (16–35) | 37 (26-41) | 24 (23-33) | 24 (20–33) | 7 (5–12) | **0.0168** | 0.7818 |
| This table is an extended version of Table 1 presenting basic demographic and non-lateralized clinical variables for 18 participants with lower right compared to left putaminal ^18^F-FE-PE2I PET binding (R < L), 15 participants with lower left compared to right putaminal ^18^F-FE-PE2I PET binding (R > L), 15 participants with symmetrical putaminal ^18^F-FE-PE2I PET binding (R = L), a combined group of participants with asymmetrical putaminal ^18^F-FE-PE2I PET binding (R ≠ L), 16 healthy elderly control subjects (HC), and statistical group comparisons. Continuous variables are reported as mean ± standard deviation if normally distributed and median (interquartile range) if non-normally distributed. Categorical variables are presented with frequency and percentage. Group comparisons are made with unpaired t-test or Mann-Whitney U test for continuous variables and Fisher’s exact test for categorical variables. P-values are uncorrected and values below 0.05 are marked in bold. H&Y = Hoehn and Yahr scale; LEDD = levodopa equivalent daily dose; NMSS = Non-Motor Symptoms Scale for Parkinson’s Disease; RBD = REM sleep behavior disorder; RBDSQ = REM Sleep Behavior Disorder Screening Questionnaire; UPDRS-III = Movement Disorder Society – Unified Parkinson’s Disease Rating Scale part III.  ^a^ Defined as systolic blood pressure > 140 mmHg or diastolic blood pressure > 90 mmHg. | | | | | | | |

| **Supplementary table 2. Striatal ^18^F-FE-PE2I PET binding** | | | | | | |
| --- | --- | --- | --- | --- | --- | --- |
|  | **R < L**  **n = 18** | **R = L**  **n = 15** | **R > L**  **n = 15** | **R ≠ L**  **n = 33** | **R = L**  **R ≠ L** | **R < L**  **R > L** |
| **Putamen, specific binding ratio** | | | | | | |
| Right | 1.70 (1.63–2.01) | 1.62 (1.47–1.91) | 3.02 (2.61–3.36) | 2.38 (1.69–3.02) | – | – |
| Left | 2.58 (2.40–2.97 | 1.54 (1.27–1.73) | 1.93 (1.60–2.21) | 2.38 (1.98–2.59) | – | – |
| Average | 2.20 (2.02–2.39) | 1.63 (1.42–1.79) | 2.50 (2.06–2.71) | 2.26 (2.03–2.70) | <**0.0001** | 0.3115 |
| **Caudate, specific binding ratio** | | | | | | |
| Right | 4.09 (3.32–4.40) | 3.19 (2.96–3.59) | 4.06 (3.86–4.70) | 4.08 (3.70–4.59) | – | – |
| Left | 4.39 (3.74–4.72) | 3.10 (2.91–3.42) | 3.78 (3.53–4.46) | 4.15 (3.59–4.64) | – | – |
| Average | 4.25 (3.47–4.47) | 3.10 (2.81–3.51) | 3.99 (3.64–4.54) | 3.99 (3.64–4.54) | <**0.0001** | 0.5736 |
| **Putamen/caudate, specific binding ratio** | | | | | | |
| Right | 0.47 (0.41–0.57) | 0.53 (0.47–0.59) | 0.66 (0.61–0.73) | 0.61 (0.47–0.67) | – | – |
| Left | 0.63 (0.55–0.87) | 0.51 (0.47–0.56) | 0.47 (0.41–0.52) | 0.54 (0.45–0.65) | – | – |
| Average | 0.55 (0.50–0.72) | 0.52 (0.46–0.57) | 0.57 (0.53–0.63) | 0.57 (0.51–0.64) | 0.0615 | 0.7748 |
| This table is an extended version of Table 2 presenting ^18^F-FE-PE2I PET binding in the putamen and caudate of 18 participants with lower right compared to left putaminal ^18^F-FE-PE2I PET binding (R < L), 15 participants with lower left compared to right putaminal ^18^F-FE-PE2I PET binding (R > L), 15 participants with symmetrical putaminal ^18^F-FE-PE2I PET binding (R = L), a combined group of participants with asymmetrical putaminal ^18^F-FE-PE2I PET binding (R ≠ L), and statistical group comparisons. All variables are continuous and non-normally distributed and reported as median (interquartile range). Group comparisons are performed using the Mann-Whitney U test. P-values are uncorrected and values below 0.05 are marked in bold. | | | | | | |

| **Supplementary table 3. Lateralized motor and non-motor assessment** | | | | | |  |  |
| --- | --- | --- | --- | --- | --- | --- | --- |
|  | **R < L**  **n = 18** | **R = L**  **n = 15** | **R > L**  **n = 15** | **R ≠ L**  **n = 33** | **HC**  **n = 16** | **R = L**  **R ≠ L** | **R < L**  **R > L** |
| **Cognition** | | | | | |  |  |
| Word recall^a^ | 6 (5–7) | 6 (3–7) | 7 (5–9) | 6 (5–8) | 8 (7–10) | 0.2679 | 0.3226 |
| Word recognition^a^ | 10 (9–10) | 10 (9–10) | 10 (10–10) | 10 (10–10) | 10 (10–10) | 0.9237 | 0.0859 |
| Line orientation^b^ | 24 (22–28)^d^ | 23 (18–26) | 26 (22–29) | 25 (22–29) | 27 (25–28) | 0.0850 | 0.7291 |
| Line bisection^c^, mm | 2 (-20–33) | 43 (17–64) | 10 (-18–28) | 8 (-18–28) | -2 (-16–38) | – | 0.5029 |
| Line bisection^e^, absolute | 22 (9–44) | 44 (26–64) | 19 (10–28) | 21 (10–40) | 21 (11–43) | **0.0106** | – |
| **Olfaction, score** | | | | | |  |  |
| Right nostril | 6 (5–9)^d^ | 6 (4–9) | 7 (5–8)^f^ | 7 (5–8) | 11 (10–13) | 0.3812 | >0.9999 |
| Left nostril | 6 (5–9)^d^ | 6 (5–7) | 7 (5–8)^f^ | 6 (5–8) | 12 (9–13) | 0.6074 | 0.8425 |
| Average | 6 (5–8)^d^ | 6 (5–8) | 7 (5–8)^f^ | 6 (5–8) | 11 (10–13) | 0.3719 | 0.8277 |
| **Skin wrinkling, score** |  |  |  |  |  |  |  |
| Right hand | 1.6 (1.0–3.0)^g^ | 1.8 (1.3–2.3)^g^ | 2.0 (1.5–2.8) | 2.0 (1.0–3.0) | 1.6 (1.3–3.3) | 0.6679 | 0.3575 |
| Left hand | 1.3 (1.0–2.0)^g^ | 1.8 (1.5–2.3)^g^ | 2.0 (1.8–3.5) | 1.8 (1.0–2.3) | 2.3 (1.3–2.5) | 0.7333 | **0.0267** |
| Average | 1.4 (1.0–2.6)^g^ | 1.9 (1.4–2.1)^g^ | 2.3 (1.9–3.0) | 1.9 (1.0–2.9) | 2.0 (1.3–2.9) | 0.9339 | 0.0848 |
| **Salivation, mg** |  |  |  |  |  |  |  |
| Right cheek | 1.89 (1.58–2.07)^d^ | 2.06 (1.92–2.17) | 2.11 (1.98–2.16) | 2.01 (1.87–2.13) | 2.07 (2.01–2.17) | 0.3476 | **0.0244** |
| Left cheek | 2.11 (1.94–2.24)^d^ | 2.06 (1.52–2.20) | 2.08 (1.88–2.11) | 2.09 (1.88–2.18) | 2.04 (1.88–2.11) | 0.8172 | 0.4331 |
| Average | 1.98 (1.53–2.08)^d^ | 2.05 (1.89–2.19) | 2.05 (1.89–2.14) | 2.01 (1.72–2.13) | 2.06 (1.94–2.11) | 0.6114 | 0.1425 |
| **Lacrimation, mm** |  |  |  |  |  |  |  |
| Right eye | 5 (4–9) | 5 (4–8) | 8 (5–14) | 6 (4–10) | 8 (5–16) | 0.1593 | 0.2090 |
| Left eye | 5 (3–8) | 5 (3–6) | 5 (4–10) | 5 (4–10) | 7 (2–15) | 0.2980 | 0.7267 |
| Average | 6 (4–8) | 5 (4–7) | 8 (4–11) | 7 (4–10) | 8 (3–14) | 0.1600 | 0.3280 |
| **Alternate Tapping Test, taps/min** | | | | | | | |
| Right | 149 (125–158) | 129 (112–147) | 149 (120–172) | 149 (125–170) | 166 (148–174) | – | – |
| Left | 132 (119–150) | 119 (109–124) | 155 (134–170) | 141 (128–156) | 154 (146–168) | – | – |
| Average | 143 (122–154) | 122 (110–134) | 156 (115–174) | 147 (122–158) | 160 (149–170) | **0.0072** | 0.1865 |
| **UPDRS-III, extremity score** | | | | | | | |
| Right | 7 (2–10) | 11 (8–16) | 14 (9–17) | 9 (5–13) | 3 (2–5) | – | – |
| Left | 14 (8–19) | 12 (10–17) | 6 (3–9) | 9 (6–14) | 3 (2–6) | – | – |
| Average | 10 (6–14) | 12 (11–14) | 9 (8–13) | 9 (6–13) | 3 (2–5) | **0.0301** | 0.8653 |
| This table is an extended version of Table 3 presenting lateralized motor and non-motor assessments in 18 participants with lower right compared to left putaminal ^18^F-FE-PE2I PET binding (R < L), 15 participants with lower left compared to right putaminal ^18^F-FE-PE2I PET binding (R > L), 15 participants with symmetrical putaminal ^18^F-FE-PE2I PET binding (R = L), a combined group of participants with asymmetrical putaminal ^18^F-FE-PE2I PET binding (R ≠ L), 17 healthy elderly control subjects (HC), and statistical group comparisons. All variables are continuous and reported as median (interquartile range). Group comparisons are performed using the Mann-Whitney U test. P-values are uncorrected and values below 0.05 are marked in bold.  ^a^ Assessed with the SCOPA-COG (Scales for Outcomes in Parkinson’s Disease-COGnition).  ^b^ Correct answers on the Benton Judgement of Line Orientation test.  ^c^ Overshoot in mm to the right on the Line Bisection test.  ^d^ Missing data for one patient.  ^e^ Absolute overshoot in mm.  ^f^ Missing data for two patients.  ^g^ Data from two patients excluded. | | | | | | | |

| **Supplementary table 4. Pupillary measurements** | | | | | | | |
| --- | --- | --- | --- | --- | --- | --- | --- |
|  | **R < L**  **n = 18** | **R = L**  **n = 15** | **R > L**  **n = 15** | **R ≠ L**  **n = 33** | **HC**  **n = 16** | **R = L**  **R ≠ L** | **R < L**  **R > L** |
| **Baseline diameter, mm** | | | | | | | |
| Right | 5.3 ± 1.0 | 4.9 ± 1.0 | 5.6 ± 1.1 | 5.5 ± 1.1 | 4.4 ± 1.1 | 0.2156 | 0.4548 |
| Left | 5.0 ± 0.8 | 4.9 ± 0.9 | 5.4 ± 1.0 | 5.2 ± 0.9 | 4.3 ± 1.1 | 0.2700 | 0.2424 |
| Average | 5.1 ± 0.6 | 4.9 ± 0.9 | 5.5 ± 1.0 | 5.3 ± 1.0 | 4.4 ± 1.1 | 0.2316 | 0.3119 |
| **Minimum diameter post-flash, mm** | | | | | | | |
| Right | 3.2 ± 0.6 | 3.0 ± 0.7 | 3.3 ± 0.8 | 3.3 ± 0.7 | 2.6 ± 0.6 | 0.2420 | 0.7613 |
| Left | 3.0 ± 0.6 | 2.9 ± 0.6 | 3.3 ± 0.8 | 3.2 ± 0.7 | 2.6 ± 0.7 | 0.3567 | 0.1818 |
| Average | 3.1 ± 0.6 | 3.0 ± 0.6 | 3.3 ± 0.8 | 3.2 ± 0.7 | 2.6 ± 0.7 | 0.2961 | 0.3700 |
| **Change from baseline, %** | | | | | | | |
| Right | 39 ± 5 | 40 ± 6 | 41 ± 7 | 40 ± 6 | 43 ± 5 | 0.9496 | 0.5284 |
| Left | 41 ± 3 | 39 ± 6 | 39 ± 7 | 40 ± 5 | 41 ± 5 | 0.8147 | 0.3413 |
| Average | 40 ± 3 | 40 ± 6 | 40 ± 6 | 40 ± 5 | 42 ± 3 | 0.8443 | 0.8319 |
| **Average constriction velocity, mm/s** | | | | | | | |
| Right | 2.5 ± 0.6 | 2.7 ± 0.5 | 2.7 ± 0.5 | 2.6 ± 0.5 | 2.5 ± 0.6 | 0.7051 | 0.3421 |
| Left | 2.5 ± 0.3 | 2.5 ± 0.5 | 2.5 ± 0.5 | 2.5 ± 0.4 | 2.3 ± 0.6 | 0.7694 | 0.8975 |
| Average | 2.5 ± 0.4 | 2.6 ± 0.5 | 2.6 ± 0.5 | 2.5 ± 0.4 | 2.4 ± 0.5 | 0.7377 | 0.6255 |
| **Maximum constriction velocity, mm/s** | | | | | | | |
| Right | 4.2 ± 0.9 | 4.3 ± 0.8 | 4.4 ± 0.9 | 4.3 ± 0.9 | 4.3 ± 0.9 | 0.9944 | 0.4787 |
| Left | 4.2 ± 0.5 | 4.3 ± 0.8 | 4.2 ± 0.9 | 4.2 ± 0.7 | 4.1 ± 0.9 | 0.5651 | 0.9429 |
| Average | 4.2 ± 0.6 | 4.3 ± 0.7 | 4.3 ± 0.8 | 4.3 ± 0.7 | 4.1 ± 0.7 | 0.7844 | 0.7251 |
| **Latency, s** | | | | | | | |
| Right | 0.2 ± 0.0 | 0.2 ± 0.0 | 0.2 ± 0.0 | 0.2 ± 0.0 | 0.2 ± 0.0 | 0.7858 | 0.4811 |
| Left | 0.2 ± 0.0 | 0.2 ± 0.0 | 0.2 ± 0.0 | 0.2 ± 0.0 | 0.2 ± 0.0 | 0.6665 | 0.2099 |
| Average | 0.2 ± 0.0 | 0.2 ± 0.2 | 0.2 ± 0.0 | 0.2 ± 0.0 | 0.2 ± 0.0 | 0.9073 | 0.2503 |
| **Average redilation speed, mm/s** | | | | | | | |
| Right | 1.0 ± 0.2 | 0.9 ± 0.2 | 1.1 ± 0.2 | 1.0 ± 0.2 | 1.0 ± 0.3 | **0.0271** | 0.3387 |
| Left | 0.8 ± 0.2 | 0.8 ± 0.2 | 1.1 ± 0.4 | 1.0 ± 0.3 | 0.8 ± 0.2 | 0.2137 | **0.0480** |
| Average | 0.9 ± 0.2 | 0.9 ± 0.2 | 1.1 ± 0.3 | 1.0 ± 0.2 | 0.8 ± 0.2 | **0.0427** | 0.1481 |
| This table is an extended version of Table 4 presenting pupillary measurements in right and left eye in 18 participants with lower right compared to left putaminal ^18^F-FE-PE2I PET binding (R < L), 15 participants with lower left compared to right putaminal ^18^F-FE-PE2I PET binding (R > L), 15 participants with symmetrical putaminal ^18^F-FE-PE2I PET binding (R = L), a combined group of participants with asymmetrical putaminal ^18^F-FE-PE2I PET binding (R ≠ L), 17 healthy elderly control subjects (HC), and statistical group comparisons. All variables are continuous and reported as mean ± standard deviation. Group comparisons are performed with unpaired t-test. P-values are uncorrected and values below 0.05 are marked in bold. | | | | | | | |

| **Supplementary table 5.** Correlations between right-left asymmetry of putamen ^18^F-FE-PE2I PET binding and right-left asymmetry in motor and non-motor measures among asymmetric patients with Parkinson’s disease | | | | |
| --- | --- | --- | --- | --- |
| Spearman r | r | 95% confidence interval | P value | P value summary |
| UPDRS-III, extremity score | 0.7270 | 0.5035 to 0.8593 | **<0.0001** | **** |
| Alternate tapping test, taps/min | -0.4525 | -0.6943 to -0.1189 | 0.0082 | ** |
| Olfaction, score | -0.1308 | -0.4777 to 0.2512 | 0.4907 | ns |
| Skin wrinkling, score | -0.2312 | -0.5489 to 0.1449 | 0.2108 | ns |
| Salivation, mg | 0.3057 | -0.0588 to 0.5983 | 0.0888 | ns |
| Lacrimation, mm | 0.0302 | -0.3259 to 0.3788 | 0.8676 | ns |
| Pupil baseline diameter, mm | -0.0811 | -0.4637 to 0.3270 | 0.6938 | ns |
| Pupil diameter post-flash, mm | -0.3252 | -0.6400 to 0.0831 | 0.1050 | ns |
| Pupil change from baseline, % | 0.2832 | -0.1289 to 0.6119 | 0.1610 | ns |
| Pupil average contriction velocity, mm/s | 0.2199 | -0.1947 to 0.5679 | 0.2803 | ns |
| Pupil maximum constriction velocity, mm/s | 0.1081 | -0.3025 to 0.4848 | 0.5993 | ns |
| Pupil latency, s | 0.1780 | -0.2363 to 0.5375 | 0.3844 | ns |
| Pupil redilation speed, mm/s | -0.5254 | -0.7716 to -0.1425 | 0.0084 | ** |
| Right-left asymmetry is defined as the absolute difference between right and left measurements. * P < 0.05, ** P < 0.01, *** P < 0.001, **** P < 0.0001. Bold marks significance after False Discovery Rate correction. | | | | |

| **Supplementary table 6.** Correlations between asymmetry index of putamen ^18^F-FE-PE2I PET binding and asymmetry index in motor and non-motor measures among asymmetric patients with Parkinson’s disease | | | | |
| --- | --- | --- | --- | --- |
| Spearman r | r | 95% confidence interval | P value | P value summary |
| UPDRS-III, extremity score | 0.7972 | 0.6185 to 0.8975 | **<0.0001** | **** |
| Alternate tapping test, taps/min | -0.3636 | -0.6348 to -0.0127 | 0.0375 | * |
| Olfaction, score | -0.1111 | -0.4621 to 0.2699 | 0.5587 | ns |
| Skin wrinkling, score | -0.1980 | -0.5241 to 0.1787 | 0.2856 | ns |
| Salivation, mg | 0.1782 | -0.1922 to 0.5041 | 0.3293 | ns |
| Lacrimation, mm | -0.1002 | -0.4373 to 0.2617 | 0.5791 | ns |
| Pupil baseline diameter, mm | -0.0886 | -0.4696 to 0.3203 | 0.6671 | ns |
| Pupil diameter post-flash, mm | -0.3757 | -0.6728 to 0.0257 | 0.0585 | ns |
| Pupil change from baseline, % | 0.2830 | -0.1291 to 0.6117 | 0.1613 | ns |
| Pupil average contriction velocity, mm/s | 0.3248 | -0.0835 to 0.6398 | 0.1054 | ns |
| Pupil maximum constriction velocity, mm/s | 0.1453 | -0.2677 to 0.5132 | 0.4788 | ns |
| Pupil latency, s | 0.2111 | -0.2036 to 0.5615 | 0.3006 | ns |
| Pupil redilation speed, mm/s | -0.2948 | -0.6316 to 0.1357 | 0.1620 | ns |
| The asymmetry index is calculated as the difference between right and left, divided by the sum of right and left. * P < 0.05, ** P < 0.01, *** P < 0.001, **** P < 0.0001. Bold marks significance after False Discovery Rate correction. | | | | |

| **Supplementary table 7**. Correlations between right-left asymmetry of putamen ^18^F-FE-PE2I PET binding and right-left asymmetry in motor and non-motor measures among all participants with Parkinson’s disease | | | | |
| --- | --- | --- | --- | --- |
| Spearman r | r | 95% confidence interval | P value | P value summary |
| UPDRS-III, extremity score | 0.8551 | 0.7504 to 0.9179 | **<0.0001** | **** |
| Alternate tapping test, taps/min | -0.4791 | -0.6765 to -0.2174 | **0.0006** | *** |
| Olfaction, score | -0.1376 | -0.4218 to 0.1712 | 0.3674 | ns |
| Skin wrinkling, score | -0.2535 | -0.5185 to 0.05591 | 0.0968 | ns |
| Salivation, mg | 0.2604 | -0.0376 to 0.5159 | 0.0771 | ns |
| Lacrimation, mm | 0.0538 | -0.2421 to 0.3405 | 0.7166 | ns |
| Pupil baseline diameter, mm | -0.0508 | -0.3773 to 0.2869 | 0.7652 | ns |
| Pupil diameter post-flash, mm | -0.2793 | -0.5601 to 0.0591 | 0.0941 | ns |
| Pupil change from baseline, % | 0.3206 | -0.0138 to 0.5905 | 0.0530 | ns |
| Pupil average contriction velocity, mm/s | 0.2739 | -0.0649 to 0.5561 | 0.1009 | ns |
| Pupil maximum constriction velocity, mm/s | 0.1885 | -0.1540 to 0.4906 | 0.2638 | ns |
| Pupil latency, s | 0.1751 | -0.1675 to 0.4800 | 0.2999 | ns |
| Pupil redilation speed, mm/s | -0.4185 | -0.6687 to -0.0832 | 0.0138 | * |
| Right-left asymmetry is defined as the absolute difference between right and left measurements. * P < 0.05, ** P < 0.01, *** P < 0.001, **** P < 0.0001. Bold marks significance after False Discovery Rate correction. | | | | |

| **Supplementary table 8**. Correlations between asymmetry index of putamen ^18^F-FE-PE2I PET binding and asymmetry index in motor and non-motor measures among all participants with Parkinson’s disease | | | | |
| --- | --- | --- | --- | --- |
| Spearman r | r | 95% confidence interval | P value | P value summary |
| UPDRS-III, extremity score | 0.9017 | 0.8277 to 0.9449 | **<0.0001** | **** |
| Alternate tapping test, taps/min | -0.4350 | -0.6451 to -0.1637 | 0.0020 | ** |
| Olfaction, score | -0.1252 | -0.4113 to 0.1834 | 0.4126 | ns |
| Skin wrinkling, score | -0.2567 | -0.5210 to 0.0526 | 0.0926 | ns |
| Salivation, mg | 0.2000 | -0.1011 to 0.4676 | 0.1776 | ns |
| Lacrimation, mm | -0.0785 | -0.3622 to 0.2186 | 0.5960 | ns |
| Pupil baseline diameter, mm | -0.0337 | -0.3625 to 0.3026 | 0.8432 | ns |
| Pupil diameter post-flash, mm | -0.3049 | -0.5790 to 0.0311 | 0.0665 | ns |
| Pupil change from baseline, % | 0.3034 | -0.03275 to 0.5779 | 0.0679 | ns |
| Pupil average contriction velocity, mm/s | 0.3292 | -0.0041 to 0.5967 | 0.0466 | * |
| Pupil maximum constriction velocity, mm/s | 0.2077 | -0.1345 to 0.5056 | 0.2174 | ns |
| Pupil latency, s | 0.1884 | -0.1542 to 0.4905 | 0.2642 | ns |
| Pupil redilation speed, mm/s | -0.2816 | -0.5729 to 0.0729 | 0.1067 | ns |
| The asymmetry index is calculated as the difference between right and left, divided by the sum of right and left. * P < 0.05, ** P < 0.01, *** P < 0.001, **** P < 0.0001. Bold marks significance after False Discovery Rate correction. | | | | |

| **Supplementary table 9**. Correlations between right-left asymmetry of caudate ^18^F-FE-PE2I PET binding and right-left asymmetry in motor and non-motor measures among asymmetric patients with Parkinson’s disease | | | | |
| --- | --- | --- | --- | --- |
| Spearman r | r | 95% confidence interval | P value | P value summary |
| UPDRS-III, extremity score | 0.7590 | 0.5410 to 0.8815 | **<0.0001** | **** |
| Alternate tapping test, taps/min | -0.2828 | -0.5909 to 0.0972 | 0.1299 | ns |
| Olfaction, score | -0.0658 | -0.4445 to 0.3329 | 0.7445 | ns |
| Skin wrinkling, score | -0.3149 | -0.6228 to 0.07744 | 0.1026 | ns |
| Salivation, mg | 0.1631 | -0.2201 to 0.5027 | 0.3890 | ns |
| Lacrimation, mm | 0.1671 | -0.2162 to 0.5058 | 0.3774 | ns |
| Pupil baseline diameter, mm | -0.2722 | -0.6233 to 0.1703 | 0.2090 | ns |
| Pupil diameter post-flash, mm | -0.3486 | -0.6724 to 0.0872 | 0.1031 | ns |
| Pupil change from baseline, % | 0.2542 | -0.1890 to 0.6114 | 0.2417 | ns |
| Pupil average contriction velocity, mm/s | 0.0695 | -0.3641 to 0.4783 | 0.7528 | ns |
| Pupil maximum constriction velocity, mm/s | -0.0870 | -0.4918 to 0.3487 | 0.6931 | ns |
| Pupil latency, s | -0.0806 | -0.4869 to 0.3544 | 0.7146 | ns |
| Pupil redilation speed, mm/s | -0.2966 | -0.6462 to 0.1559 | 0.1801 | ns |
| Right-left asymmetry is defined as the absolute difference between right and left measurements. * P < 0.05, ** P < 0.01, *** P < 0.001, **** P < 0.0001. Bold marks significance after False Discovery Rate correction. | | | | |

| **Supplementary table 10**. Correlations between asymmetry index of caudate ^18^F-FE-PE2I PET binding and asymmetry index in motor and non-motor measures among asymmetric patients with Parkinson’s disease | | | | |
| --- | --- | --- | --- | --- |
| Spearman r | r | 95% confidence interval | P value | P value summary |
| UPDRS-III, extremity score | 0.6989 | 0.4437 to 0.8493 | **<0.0001** | **** |
| Alternate tapping test, taps/min | -0.2627 | -0.5766 to 0.1187 | 0.1607 | ns |
| Olfaction, score | -0.0855 | -0.4602 to 0.3151 | 0.6717 | ns |
| Skin wrinkling, score | -0.2669 | -0.5897 to 0.1293 | 0.1697 | ns |
| Salivation, mg | 0.1328 | -0.2494 to 0.4792 | 0.4841 | ns |
| Lacrimation, mm | -0.0383 | -0.4025 to 0.3364 | 0.8408 | ns |
| Pupil baseline diameter, mm | -0.2460 | -0.6059 to 0.1974 | 0.2578 | ns |
| Pupil diameter post-flash, mm | -0.4407 | -0.7279 to -0.0219 | 0.0353 | * |
| Pupil change from baseline, % | 0.3086 | -0.1314 to 0.6471 | 0.1519 | ns |
| Pupil average contriction velocity, mm/s | 0.1497 | -0.2916 to 0.5385 | 0.4953 | ns |
| Pupil maximum constriction velocity, mm/s | 0.0178 | -0.4082 to 0.4374 | 0.9358 | ns |
| Pupil latency, s | -0.0539 | -0.4662 to 0.3776 | 0.8070 | ns |
| Pupil redilation speed, mm/s | -0.3111 | -0.6554 to 0.1402 | 0.1587 | ns |
| The asymmetry index is calculated as the difference between right and left, divided by the sum of right and left. * P < 0.05, ** P < 0.01, *** P < 0.001, **** P < 0.0001. Bold marks significance after False Discovery Rate correction. | | | | |

| **Supplementary table 11**. Correlations between right-left asymmetry of caudate ^18^F-FE-PE2I PET binding and right-left asymmetry in motor and non-motor measures among all patients with Parkinson’s disease | | | | |
| --- | --- | --- | --- | --- |
| Spearman r | r | 95% confidence interval | P value | P value summary |
| UPDRS-III, extremity score | 0.8360 | 0.7145 to 0.9085 | **<0.0001** | **** |
| Alternate tapping test, taps/min | -0.3700 | -0.6043 to -0.0780 | 0.0123 | * |
| Olfaction, score | -0.1547 | -0.4455 to 0.1656 | 0.3279 | ns |
| Skin wrinkling, score | -0.2813 | -0.5486 to 0.0382 | 0.0748 | ns |
| Salivation, mg | 0.2131 | -0.0947 to 0.4837 | 0.1599 | ns |
| Lacrimation, mm | 0.1184 | -0.1901 to 0.4056 | 0.4387 | ns |
| Pupil baseline diameter, mm | -0.0036 | -0.3505 to 0.3442 | 0.9839 | ns |
| Pupil diameter post-flash, mm | -0.1299 | -0.4566 to 0.2278 | 0.4642 | ns |
| Pupil change from baseline, % | 0.2427 | -0.1143 to 0.5442 | 0.1667 | ns |
| Pupil average contriction velocity, mm/s | 0.1940 | -0.1644 to 0.5072 | 0.2715 | ns |
| Pupil maximum constriction velocity, mm/s | 0.1586 | -0.1997 to 0.4796 | 0.3702 | ns |
| Pupil latency, s | 0.0316 | -0.3192 to 0.3748 | 0.8592 | ns |
| Pupil redilation speed, mm/s | -0.3016 | -0.5954 to 0.0633 | 0.0934 | ns |
| Right-left asymmetry is defined as the absolute difference between right and left measurements. * P < 0.05, ** P < 0.01, *** P < 0.001, **** P < 0.0001. Bold marks significance after False Discovery Rate correction. | | | | |

| **Supplementary table 12**. Correlations between asymmetry index of caudate ^18^F-FE-PE2I PET binding and asymmetry index in motor and non-motor measures among all participants with Parkinson’s disease | | | | |
| --- | --- | --- | --- | --- |
| Spearman r | r | 95% confidence interval | P value | P value summary |
| UPDRS-III, extremity score | 0.7697 | 0.6096 to 0.8695 | **<0.0001** | **** |
| Alternate tapping test, taps/min | -0.3942 | -0.6219 to -0.1050 | 0.0074 | ** |
| Olfaction, score | -0.2077 | -0.4884 to 0.1119 | 0.1868 | ns |
| Skin wrinkling, score | -0.2697 | -0.5398 to 0.0508 | 0.0882 | ns |
| Salivation, mg | 0.2055 | -0.1025 to 0.4776 | 0.1756 | ns |
| Lacrimation, mm | -0.0358 | -0.3338 to 0.2688 | 0.8156 | ns |
| Pupil baseline diameter, mm | 0.0237 | -0.3264 to 0.3680 | 0.8942 | ns |
| Pupil diameter post-flash, mm | -0.1630 | -0.4830 to 0.1954 | 0.3569 | ns |
| Pupil change from baseline, % | 0.2664 | -0.0892 to 0.5618 | 0.1278 | ns |
| Pupil average contriction velocity, mm/s | 0.2448 | -0.1121 to 0.5457 | 0.1629 | ns |
| Pupil maximum constriction velocity, mm/s | 0.2070 | -0.1512 to 0.5172 | 0.2401 | ns |
| Pupil latency, s | 0.0306 | -0.3201 to 0.3740 | 0.8635 | ns |
| Pupil redilation speed, mm/s | -0.2709 | -0.5734 to 0.0966 | 0.1337 | ns |
| The asymmetry index is calculated as the difference between right and left, divided by the sum of right and left. * P < 0.05, ** P < 0.01, *** P < 0.001, **** P < 0.0001. Bold marks significance after False Discovery Rate correction. | | | | |

| **Supplementary table 13**. Correlations between non-negative right minus left putamen ^18^F-FE-PE2I PET binding and demographic, non-lateralized non-motor variables, averaged striatal ^18^F-FE-PE2I PET binding, and averaged lateralized motor and non-motor variables among asymmetric patients with Parkinson’s disease | | | | | |
| --- | --- | --- | --- | --- | --- |
| Spearman r | | r | 95% confidence interval | P value | P value summary |
| Demographic and non-lateralized non-motor variables | | | | |  |
|  | Age | -0.1658 | -0.4898 to 0.1984 | 0.3565 | ns |
|  | RBDSQ, score | 0.0012 | -0.3516 to 0.3536 | 0.9948 | ns |
|  | NMSS, score | -0.1774 | -0.4988 to 0.1869 | 0.3234 | ns |
|  | Maximum systolic drop, mmHg | 0.1901 | -0.1803 to 0.5133 | 0.2974 | ns |
|  | Maximum diastolic drop, mmHg | 0.2372 | -0.1321 to 0.5487 | 0.1911 | ns |
| Averaged striatal ^18^F-FE-PE2I PET binding | | | |  |  |
|  | Putamen, average SBR | 0.5066 | 0.1875 to 0.7290 | 0.0026 | ** |
|  | Caudate, average SBR | 0.1017 | -0.2787 to 0.4545 | 0.5929 | ns |
|  | Putamen/caudate, average SBR | 0.3264 | -0.04955 to 0.6213 | 0.0784 | ns |
| Averaged lateralized motor and non-motor variables | | | |  |  |
|  | SCOPA-COG, 1st recall | 0.2642 | -0.09747 to 0.5643 | 0.1373 | ns |
|  | SCOPA-COG, 2nd recall | 0.1775 | -0.1868 to 0.4989 | 0.3229 | ns |
|  | SCOPA-COG, 3rd recall | 0.2141 | -0.1498 to 0.5269 | 0.2315 | ns |
|  | SCOPA-COG, delayed recall | 0.1818 | -0.1825 to 0.5022 | 0.3114 | ns |
|  | SCOPA-COG, recognition | 0.3576 | 0.005696 to 0.6307 | 0.0410 | * |
|  | Line orientation, score | 0.2314 | -0.1382 to 0.5444 | 0.2026 | ns |
|  | Line bisection, mm | -0.0551 | -0.3999 to 0.3034 | 0.7607 | ns |
|  | Olfaction, score | 0.2325 | -0.1503 to 0.5547 | 0.2163 | ns |
|  | Skin wrinkling, score | -0.1293 | -0.4710 to 0.2461 | 0.4881 | ns |
|  | Salivation, mg | 0.2423 | -0.1268 to 0.5525 | 0.1815 | ns |
|  | Lacrimation, mm | 0.3491 | -0.003956 to 0.6248 | 0.0464 | * |
|  | UPDRS-III, score | -0.1937 | -0.5113 to 0.1706 | 0.2802 | ns |
|  | Alternate tapping test, taps/min | 0.5110 | 0.1932 to 0.7318 | 0.0024 | ** |
| Right-left asymmetry is defined as the absolute difference between right and left measurements. Lateralized variables are presented as averages calculated as right plus left divided by two. * P < 0.05, ** P < 0.01, *** P < 0.001, **** P < 0.0001. Bold marks significance after False Discovery Rate correction. NMSS = Non-Motor Symptoms Scale for Parkinson’s Disease; RBD = REM sleep behavior disorder; RBDSQ = REM Sleep Behavior Disorder Screening Questionnaire; SBR, specific binding ratio; SCOPA-COG (Scales for Outcomes in Parkinson’s Disease-COGnition); UPDRS-III = Movement Disorder Society – Unified Parkinson’s Disease Rating Scale part III. | | | | | |

| **Supplementary table 14**. Correlations between non-negative right minus left putamen ^18^F-FE-PE2I PET binding and demographic, non-lateralized non-motor variables, averaged striatal ^18^F-FE-PE2I PET binding, and averaged lateralized motor and non-motor variables among all patients with Parkinson’s disease | | | | | |
| --- | --- | --- | --- | --- | --- |
| Spearman r | | r | 95% confidence interval | P value | P value summary |
| Demographic and non-lateralized non-motor variables | | | | | |
|  | Age, years | -0.3858 | -0.6092 to -0.1057 | 0.0068 | ** |
|  | RBDSQ, score | -0.2274 | -0.4871 to 0.06931 | 0.1202 | ns |
|  | NMSS, score | -0.1666 | -0.4374 to 0.1319 | 0.2577 | ns |
|  | Maximum systolic drop, mmHg | 0.2960 | 0.0009 to 0.5436 | 0.0434 | * |
|  | Maximum diastolic drop, mmHg | 0.1337 | -0.1681 to 0.4126 | 0.3704 | ns |
| Averaged striatal ^18^F-FE-PE2I PET binding | | | | | |
|  | Putamen, average SBR | 0.7706 | 0.6175 to 0.8674 | **<0.0001** | **** |
|  | Caudate, average SBR | 0.4867 | 0.2168 to 0.6874 | **0.0007** | *** |
|  | Putamen/caudate, average SBR | 0.3783 | 0.08647 to 0.6103 | 0.0104 | * |
| Averaged lateralized motor and non-motor variables | | | | | |
|  | SCOPA-COG, 1st recall | 0.3606 | 0.07667 to 0.5905 | 0.0118 | * |
|  | SCOPA-COG, 2nd recall | 0.2104 | -0.08700 to 0.4734 | 0.1512 | ns |
|  | SCOPA-COG, 3rd recall | 0.2073 | -0.09022 to 0.4708 | 0.1574 | ns |
|  | SCOPA-COG, delayed recall | 0.1997 | -0.09805 to 0.4647 | 0.1735 | ns |
|  | SCOPA-COG, recognition | 0.2302 | -0.06632 to 0.4893 | 0.1155 | ns |
|  | Line orientation, score | 0.2898 | -0.005880 to 0.5389 | 0.0482 | * |
|  | Line bisection, mm | -0.3289 | -0.5666 to -0.04081 | 0.0224 | * |
|  | Olfaction, score | 0.2580 | -0.04740 to 0.5192 | 0.0871 | ns |
|  | Skin wrinkling, score | -0.0536 | -0.3530 to 0.2557 | 0.7296 | ns |
|  | Salivation, mg | 0.0189 | -0.2778 to 0.3123 | 0.8996 | ns |
|  | Lacrimation, mm | 0.3116 | 0.0215 to 0.5533 | 0.0311 | * |
|  | UPDRS-III, score | -0.3398 | -0.5748 to -0.05297 | 0.0181 | * |
|  | Alternate tapping test, taps/min | 0.4945 | 0.2367 to 0.6873 | **0.0004** | *** |
| Right-left asymmetry is defined as the absolute difference between right and left measurements. Lateralized variables are presented as averages calculated as right plus left divided by two. * P < 0.05, ** P < 0.01, *** P < 0.001, **** P < 0.0001. Bold marks significance after False Discovery Rate correction. NMSS = Non-Motor Symptoms Scale for Parkinson’s Disease; RBD = REM sleep behavior disorder; RBDSQ = REM Sleep Behavior Disorder Screening Questionnaire; SBR, specific binding ratio; SCOPA-COG (Scales for Outcomes in Parkinson’s Disease-COGnition); UPDRS-III = Movement Disorder Society – Unified Parkinson’s Disease Rating Scale part III. | | | | | |
